# Automatic Clinical Assessment of Swallowing Behavior and Diagnosis of Silent Aspiration Using Wireless Multimodal Wearable Electronics

**DOI:** 10.1002/advs.202404211

**Published:** 2024-07-09

**Authors:** Beomjune Shin, Sung Hoon Lee, Kangkyu Kwon, Yoon Jae Lee, Nikita Crispe, So‐Young Ahn, Sandeep Shelly, Nathaniel Sundholm, Andrew Tkaczuk, Min‐Kyung Yeo, Hyojung J. Choo, Woon‐Hong Yeo

**Affiliations:** ^1^ George W. Woodruff School of Mechanical Engineering Georgia Institute of Technology Atlanta GA 30332 USA; ^2^ Wearable Intelligent Systems and Healthcare Center (WISH Center) Institute for Matter and Systems Georgia Institute of Technology Atlanta GA 30332 USA; ^3^ School of Electrical and Computer Engineering Georgia Institute of Technology Atlanta GA 30332 USA; ^4^ Wallace H. Coulter Department of Biomedical Engineering Georgia Institute of Technology and Emory University School of Medicine Atlanta GA 30332 USA; ^5^ Department of Rehabilitation Medicine Chungnam National University School of Medicine Daejeon 35015 Republic of Korea; ^6^ Department of Otolaryngology–Head and Neck Surgery School of Medicine Emory University Atlanta GA 30322 USA; ^7^ Department of Pathology Chungnam National University School of Medicine Daejeon 35015 Republic of Korea; ^8^ Department of Cell Biology School of Medicine Emory University Atlanta GA 30322 USA; ^9^ Parker H. Petit Institute for Bioengineering and Biosciences Institute for Robotics and Intelligent Machines Georgia Institute of Technology Atlanta GA 30332 USA

**Keywords:** dysphagia, kirigami structure, machine learning, silent aspiration, swallowing disorder, wearable electronics

## Abstract

Dysphagia is more common in conditions such as stroke, Parkinson's disease, and head and neck cancer. This can lead to pneumonia, choking, malnutrition, and dehydration. Currently, the diagnostic gold standard uses radiologic imaging, the videofluoroscopic swallow study (VFSS); however, it is expensive and necessitates specialized facilities and trained personnel. Although several devices attempt to address the limitations, none offer the clinical‐grade quality and accuracy of the VFSS. Here, this study reports a wireless multimodal wearable system with machine learning for automatic, accurate clinical assessment of swallowing behavior and diagnosis of silent aspirations from dysphagia patients. The device includes a kirigami‐structured electrode that suppresses changes in skin contact impedance caused by movements and a microphone with a gel layer that effectively blocks external noise for measuring high‐quality electromyograms and swallowing sounds. The deep learning algorithm offers the classification of swallowing patterns while diagnosing silent aspirations, with an accuracy of 89.47%. The demonstration with post‐stroke patients captures the system's significance in measuring multiple physiological signals in real‐time for detecting swallowing disorders, validated by comparing them with the VFSS. The multimodal electronics can ensure a promising future for dysphagia healthcare and rehabilitation therapy, providing an accurate, non‐invasive alternative for monitoring swallowing and aspiration events.

## Graphical abstract

This article reports a wireless multimodal wearable system with machine learning for automatic, accurate clinical assessment of swallowing behavior and diagnosis of silent aspirations from dysphagia patients.

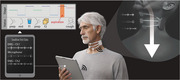

## 1 Introduction

Dysphagia is estimated to affect ≈9.44 million patients annually in the United States due to various underlying conditions,^[^ ^1^, ^2^, ^3^ ^]^ and can have an incidence as high as 80% in populations like brainstem stroke.^[^ ^4^ ^]^ Dysphagia is often underdiagnosed and undertreated where quality of life is negatively impacted and harmful consequences such as aspiration pneumonia,^[^ ^5^ ^]^ choking, malnutrition, dehydration, and even death can occur.^[^ ^6^ ^]^ Aspiration, the phenomenon of a bolus entering the airway, is very clinically relevant as it can lead to acute respiratory distress and chronically exacerbate respiratory fatigue.^[^ ^7^ ^]^ Patients with dysphagia have twice the risk of developing pneumonia compared to those without dysphagia.^[^ ^8^ ^]^ This higher risk of pneumonia is attributed to swallowing disorder symptoms that are more common among individuals with dysphagia, including penetration, aspiration, or silent aspiration. Dysphagia is identified in half of the stroke survivors^[^ ^9^ ^]^ and two‐thirds of stroke patients with dysphagia suffer from silent aspiration.^[^ ^10^ ^]^ Silent aspiration is particularly dangerous compared to aspiration that presents with overt symptoms such as coughing, because it occurs without explicit symptoms. This makes it challenging to detect at the bedside due to a reduction or complete lack of a patient's protective responses via sensory reflexes^[^ ^11^ ^]^ and the absence of convenient screening tools. Hence, repetitive swallowing evaluations are often necessary for certain populations like those post‐stroke to safely rehabilitate and mitigate aspiration risk when oral alimentation is possible. Through the evaluations, patients get guided with the consistency of the food that does not pose a risk of silent aspiration, thereby ensuring they receive nutritional supply during the rehabilitation process.

The flexible endoscopic evaluation of swallowing^[^ ^12^ ^]^ and X‐ray‐based VFSS are the main instrumental assessments to investigate swallowing dysfunction and guide treatment of dysphagia.^[^ ^13^ ^]^ Among these, VFSS best visualizes all stages of swallowing and can effectively capture silent aspiration. It is considered the standard for diagnosing and characterizing the severity of oropharyngeal dysphagia. However, this is a specialized test that does have some limitations: 1) patients must visit a facility with fluoroscopic capabilities, such as a hospital, 2) patients are exposed to ionizing radiation, 3) barium, the frequently used contrast agent, has potential side effects such as intolerance, allergic reaction, and itself is harmful if aspirated, and 4) highly trained personnel must participate in the interpretation of the data.^[^ ^14^ ^]^ To address these issues, attempts have been made to monitor deglutition by utilizing the bio‐signals that occur during swallowing, such as sounds,^[^ ^15^, ^16^, ^17^ ^]^ electromyograms (EMG),^[^ ^18^, ^19^, ^20^, ^21^, ^22^, ^23^ ^]^ acceleration of the body,^[^ ^24^, ^25^, ^26^, ^27^ ^]^ and pressure changes.^[^ ^28^, ^29^ ^]^ Since swallowing is a motion created by the sequential movement of complex muscles, monitoring a single signal can only provide indirect information about the swallowing state, such as swallow duration or intensity. Therefore, research is continuously being conducted to achieve more precise measurements by simultaneously utilizing various modalities.^[^ ^22^, ^30^ ^]^ Additionally, recent studies aim to analyze the measured signals through machine learning to provide clinical information.^[^ ^30^, ^31^, ^32^, ^33^ ^]^ Along with this, efforts are being made to eliminate the discomforts of conventional swallowing monitoring methods by developing wearable monitoring systems.^[^ ^34^ ^]^ Nonetheless, it is still challenging to find wearable systems that can provide clinically relevant information, such as silent aspiration. Among the limitations is the ability to directly assist in risk assessments that often can arise from abnormal swallowing phenomena experienced by dysphagia patients. To alleviate the discomfort experienced by dysphagia patients due to the limitations of current methods for prolonged or continuous swallowing monitoring, a device that can provide specific clinical assessment regarding silent aspiration depending on food consistency is required.

In this paper, we introduce a multimodal wearable system that offers an automated classification of the swallowing status, including silent aspiration, which is an entity that necessitates early and accurate clinical detection. The device comprehensively assesses swallowing patterns by seamlessly integrating dual‐channel EMG with a microphone. Acoustic monitoring from the microphone provides information regarding glottic adduction and competence during deglutition with its sequence with two‐channel EMG, which offers a more comprehensive motor profile of the swallowing mechanism and sheds light on the intricate synchronization sequence of swallowing. Particularly, by applying a kirigami pattern^[^ ^35^ ^]^ that can isolate strain to the EMG patches, which are highly influenced by the contact state of the electrodes, the wearable patch enables the detection of high‐quality signals. Thus, this device, collecting data with VFSS, allows using clinically evaluated data for machine learning. With these evaluations and the addition of a supplemented machine learning component, this research proposes an at‐home solution that can monitor the overall swallowing actions of the subject with the hope of distinguishing when silent aspiration occurs without the need for VFSS automatically. A sensitive wearable swallowing monitor that detects silent aspiration events would encourage patients with dysphagia to perform repetitive monitoring of swallowing and safe rehabilitation at home, reducing the need for hospital visits and potentially liberalizing their diet by reducing the need for supplemental nutrition.

## 2 Results

### 2.1 Design, Architecture, and Swallowing Detection Process Using a Wireless Multimodal Wearable Electronic System

**Figure 1:**
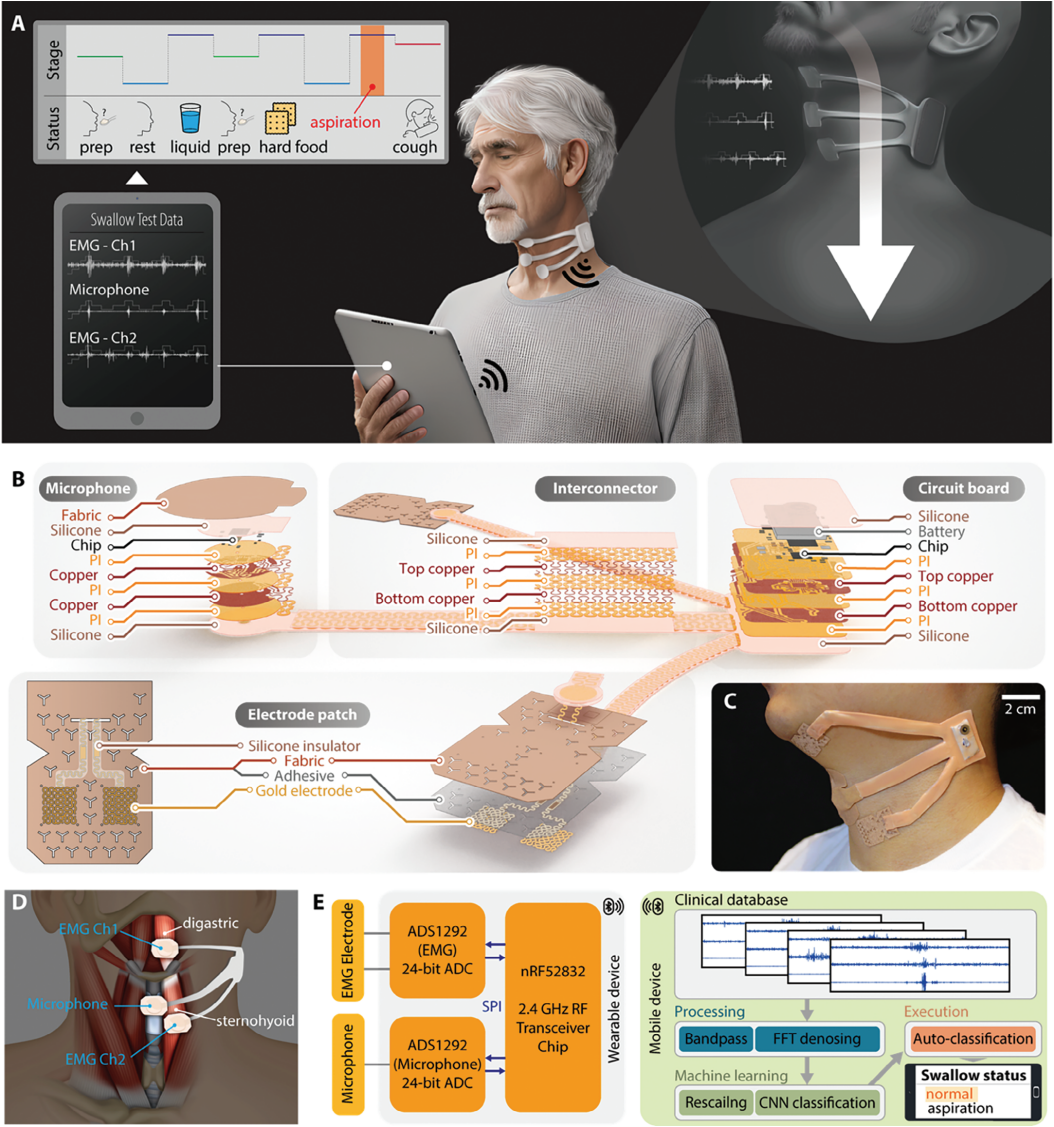
Design, architecture, and swallowing detection process using a wireless multimodal wearable electronic system. A) Illustration of the portable wireless wearable system mounted on the neck to detect swallowing behavior and abnormality without special facilities and trained personnel. B) Exploded views of the device, including two EMG electrodes, a microphone, a wireless circuit, and stretchable interconnectors encapsulated by a soft membrane. C) Photo of the wearable device mounted on the skin with three extended sensing pads. D) Schematic anatomy of the human neck related to the swallowing movements and the locations where the sensors are positioned. E) Flowchart showing the process from physiological signal detection to data classification. Signals are acquired from EMG electrodes and a microphone to a digitized analog‐to‐digital converter and sent via Bluetooth for deep learning analysis, automatic swallowing classification, and abnormal aspiration detection.

**Figure** **1**A presents an overview of a multimodal wearable electronic system. This device consists of two electrodes for measuring the EMG signals, a microphone to collect acoustic signals, and a circuit capable of processing the acquired data and transmitting it to a mobile device (Video S1, Supporting Information). Presented in Figure 1B is an in‐depth depiction of the structure of the sensors, circuit, and components comprising the entire multimodal wearable system along with stretchable and bendable interconnects that conform to neck curvature with minimal change in hardware resistance (Figure S1, Supporting Information). The device was designed to collect data concurrently with the VFSS exam (Figure S2, Supporting Information), allowing for the acquisition of accurately labeled data. The placement of the circuit at the back of the neck prevents interference along the path of bolus transit during the VFSS. The main flexible circuit board (fPCB) consists of a multilayered flexible circuit with components including a Bluetooth‐based wireless module and a rechargeable battery (Figure S3 and Table S1, Supporting Information), enabling the processing and transmission of the measured data wirelessly and continuously. All materials that have the potential to come in direct contact with the skin are encapsulated with a biocompatible silicone material (Figure S4, Supporting Information). The microphone employs a micro‐electronic mechanical system (MEMS) microphone with a preamplifier for the chip components, chosen for their compact diaphragm that is well‐suited for sound recording with sound amplification. In particular, the gel layer added between the microphone and the skin adheres conformally to the neck, where the surface curvature continuously changes during the swallowing process, effectively blocking the intrusion of external noise. The EMG electrodes are seamlessly integrated into a soft fabric composite made of non‐woven polyurethane and medical‐grade silicone adhesive. The application of a Y‐shaped kirigami pattern allows it to maintain secure adhesion, even during movements of the neck having intricate curves. Beneath the silicone adhesive lies a nanomembrane gold electrode with a serpentine structure, minimizing signal impact due to patch deformation and enhancing skin contact. Extra serpentine interconnects connecting the electrodes serve as stretchable connectors to the circuit and are insulated with silicone to avoid extra contact with the skin that could lead to unwanted noise. Detailing the intricacies of the multimodal wearable system, Figure 1C shows the actual device worn on the left side of the neck, adept at capturing EMG signals from specific muscle groups engaged in the deglutition. Positioned anteriorly on the neck overlying the cricothyroid membrane, the stethoscope captures sounds produced during deglutition which include glottic adduction, pharyngoesophageal segment opening, nuanced sounds of bolus transit, and instances of aspiration. Additionally, it provides swallowing monitoring for patients with swallowing dysfunctions without any skin irritation (Figure S5, Supporting Information). In addition to nanomembrane electrodes, the multimodal wearable swallowing monitor comes with a MEMS microphone equipped with a preamplifier circuit to collect and amplify microphone data. This microphone is crucial for capturing precise sound data related to swallowing. Expanding upon the capability of the microphone is an integrated preamplifier circuit in which the captured sound signals are enhanced and relayed with minimal noise interference. This is essential for achieving accurate and clear data readings. Expanding the comprehensive overview, Figure 1D demonstrates the anatomical intricacies of the human neck linked with swallowing movements and meticulously pinpoints the strategic placement of the sensors. The upper EMG electrode (indicated as EMG channel 1) is placed on the suprahyoid musculature overlying the digastric muscle, while the lower EMG electrode (indicated as EMG channel 2) electrode, infrahyoid overlying the sternohyoid muscle. Due to the overlapping of multiple muscles in the suprahyoid and infrahyoid regions, there is a possibility that signals from various muscles may blend. To minimize this effect and capture the movements of the digastric and sternohyoid muscles, electrodes were positioned in alignment with the muscle fiber directions of those muscles. The Microphone is also placed overlying the cricothyroid membrane between the thyroid and cricoid cartilages. Illustrating the comprehensive data analysis process, Figure 1E unveils the data analysis flowchart, elucidating the comprehensive steps from data acquisition to auto‐classification by execution of the machine learning model, ensuring precise detection and categorization of swallowing anomalies. The activity of the target muscles during swallowing and the sound data generated as a result is collected through the device and sent to a designated analog‐to‐digital converter (ADC) communicated via serial peripheral interface protocol. The packets of data are then wirelessly transmitted to a mobile app using Bluetooth Low Energy (BLE) technology (Figure S6, Supporting Information). The preprocessed data is analyzed using convolutional neural network (CNN) and long short‐term memory (LSTM) hybrid algorithms. These algorithms are capable of extracting features of data patterns and performing assessments on the overall swallowing status. In particular, it can classify the consistency of swallowed food and identify silent aspiration, which can be challenging to detect without VFSS. As a result, the multimodal wearable swallowing monitor not only enables the auto‐classification of swallowing status without the need for a hospital visit but also offers the capability to monitor silent aspiration, a symptom that can become fatal.

### 2.2 Kirigami‐Structured Device for Enhanced Conformal Contact and More Consistent Signal Detection

**Figure 2:**
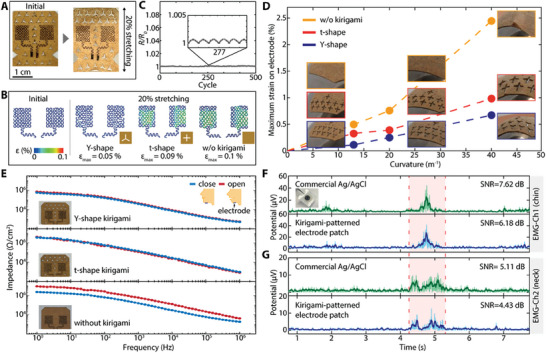
Kirigami‐structured device for enhanced conformal contact and more consistent signal detection. A) Photo of fabric with kirigami cuts for embedding serpentine electrodes, designed for enhanced strain distribution and better skin contact. B) FEA results showing the comparison of strain distribution in electrodes with different kirigami patterns under tension, highlighting the best performance from the Y‐shape. C) Negligible electrical resistance changes of the device under 20% tension capturing the strain‐damping effect of the Y‐shaped kirigami patterns. D) Comparison of the maximum strain applied to the electrode according to the perforated pattern when attached to the patch spherical dummy (inset: photos of the actual patch attachment state). E) Skin‐electrode contact impedance on the chin demonstrating the stable contact of the patch despite jaw movement (open and close), showing the advantages of kirigami patterns for consistent signal recording. F,G) EMG signal comparison from the digastric and sternohyoid muscles during swallowing action, using the kirigami patch and commercial Ag/AgCl electrodes. The results show similar data quality and SNR, although the commercial one shows a slightly higher signal due to a much bigger electrode contact area.

Surface EMG does not directly measure signals from muscles but measures the electrical potential from the surface of the skin. Thus, it is greatly influenced by the contact state between the skin and the electrode. The commercially available Ag/AgCl gel electrodes are extensively used for EMG measurements due to their high conductivity and stability. Nevertheless, they may cause problems, such as skin irritation by the gel.^[^ ^36^, ^37^ ^]^ Alternatively, dry electrodes can be used, albeit with slight performance degradation.^[^ ^38^, ^39^, ^40^ ^]^ However, the neck, where the target muscle for our EMG signal measurement is located, is a body part that easily forms wrinkles with aging, has complex curvatures, and undergoes large deformations during the swallowing movement. Therefore, a method is required to ensure that the electrode can maintain stable conformal contact with the skin at the precise location throughout the duration of the monitoring. In previous studies introducing dry electrodes, various methods have been used to ensure that the electrodes can achieve conformal contact, such as designing the electrodes in a thin serpentine structure.^[^ ^41^ ^]^ The serpentine electrodes, being thin and favorable for stretching, can adapt to the curvatures of the skin, enabling conformal contact. Furthermore, when these electrodes are stretched, the resistance changes very minimally, thus minimizing signal differences caused by deformation.^[^ ^41^, ^42^, ^43^ ^]^ However, when a patch is used for adhesion, relative motion occurs between the skin and the electrode embedded in the adhesive surface of the patch during movement. Particularly in situations with complex curvature or movement that causes the patch to shift, it is challenging to ensure stable conformal contact. To overcome these issues, we developed a serpentine‐structured electrode embedded in a patch applied with a kirigami pattern. The electrode was manufactured by laser processing an Au layer of 15 nm thickness, which was deposited on a PI film of 7.5 µm thickness into a serpentine shape. **Figure** **2**A shows this serpentine electrode is embedded in a fabric patch with a kirigami pattern. The kirigami pattern allows the patch to first deform around the edges of the cut parts when the patch is stretched, preventing the strain from spreading to the area where the electrode is located. Depending on the perforated shape, the amount of strain applied to the electrode is reduced compared to when it is not perforated (Figure 2B). Finite element analysis (FEA) results show different maximum strains applied to the electrode under 20% tension: 0.05% for the y‐shaped, 0.09% for the t‐shaped, and 0.1% when there is no pattern (Figure 2B). Due to the strain‐damping effect of the kirigami pattern, even if 25% stretching is repeatedly applied to the y‐shaped patch as shown in Figure 2C, the resistance of the electrode only changes by 0.06% compared to the initial state. In contrast, the electrode without a kirigami pattern shows a change of 0.15% under the same conditions (Figures S7 and S8, Supporting Information). Also, the perforated pattern can enhance the conformal contact of the patch on a surface with a large curvature, helping the electrode underneath to make better contact with the skin. The insets of Figure 2D show pictures of the patch attached to a sphere model with various curvatures. Compared to the patch with the kirigami pattern that sticks conformally regardless of the curvature, the patch without the kirigami pattern lifts as the curvature increases. The graph in Figure 2D plots the FEA results for the maximum strain applied to the electrode when the patch is attached to a curved surface. As the curvature of the substrate increases, greater strain is applied to the electrode, and especially in the case without kirigami, the change is much greater compared to the case with kirigami. Based on these features, the perforated patch can maintain a constant contact state with the skin even when there is movement around the neck. We measured the impedance to observe changes in the contact state between the skin and the electrode due to movement. Each electrode was attached under the chin (digastric muscle), and the impedance was measured from 1 to 10^6^ Hz when the mouth was closed and open. In the case of having a kirigami pattern, the measured impedance shows no difference regardless of the motion state of the mouth, while the impedance changes without a kirigami pattern (Figure 2E). This indicates that the kirigami patch can prevent potential changes in the contact state between the electrode and the skin that result from the deformation of the patch that can occur during swallowing movements. We compared the EMG signals from the chin (digastric muscle) and neck (sternohyoid muscle) during the swallowing action using the kirigami patch and the commercial Ag/AgCl electrode. As shown in Figure 2F,G, the EMG signals measured with the patch exhibit a similar pattern to those measured with commercial electrodes, as featured by the positions of the peaks. From the chin, we were able to obtain a signal‐to‐noise ratio (SNR) of 7.62 dB from the commercial electrode and 6.18 dB from the kirigami patch (Figure 2F), and from the neck, they were measured as 5.11 and 4.43 dB, respectively (Figure 2G). Consequently, the serpentine‐structured nanomembrane electrode minimizes the resistance change due to the strain, and the kirigami patch maintains conformal contact at various curvatures. With these advantages, the wearable patch can stably measure EMG signals when the swallowing action is performed with many movements.

### 2.3 Performance of a Soft Material‐Enabled Wearable Sensor for Sound Detection

**Figure 3:**
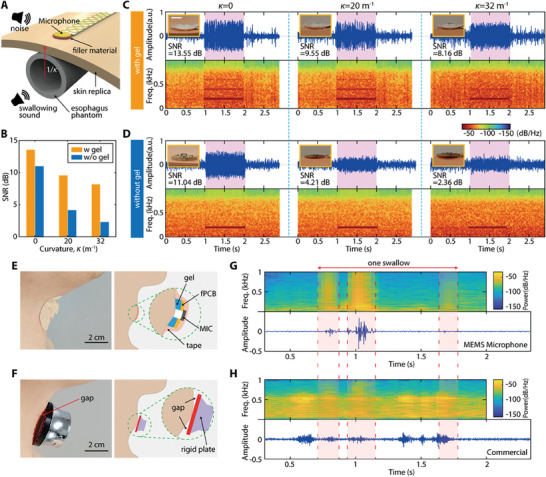
Performance of a soft material‐enabled wearable sensor for sound detection. A) Schematic of a testing setup, showing the microphone consistently exposed to white noise and a 200 Hz chirp to simulate a hospital environment while measuring swallowing sound profiles through an artificial esophagus. B) Graph showing SNR values according to the curvature of the skin replica, comparing the presence and absence of a gel layer for sound isolation. C,D) 2D plots and spectrograms depicting the placement of the microphone on a skin replica, illustrating waveform collection with a gel layer and the detrimental effects on SNR from gaps without the gel on increased curvatures. Scale bar: 5 mm. E) Application of the microphone with gel on the neck, demonstrating optimal conformity over the thyroid cartilage for efficient sound capture. F) A rigid commercial device on the skin with an air gap when attached to the skin, introducing ambient noise intrusion. G,H) Comparative spectrograms showing the soft platform‐based microphone (G) and the commercial device (H), demonstrating clear differences in swallowing event detection and sound quality. The rigid one has many motion‐related noise disturbances in signal recording.

In the design of an advanced multimodal wearable swallowing monitor, attention to detail is crucial, particularly when it comes to integrating a microphone to collect accurate swallowing timings. The MEMS microphone is a core component for capturing sound with high fidelity in wearables, especially due to its small form factor.^[^ ^44^ ^]^ For optimal performance, it is crucial to maintain a consistent air gap between the skin and the microphone diaphragm. In this study, we utilized a gel layer as a filler material between the microphone and skin to preserve the air gap. The gel layer provides a secure fit, reducing the recording of external noise and diminishing motion noise, which can be caused by the vertical movement of the thyroid cartilage during swallowing. **Figure** **3**A shows a schematic of the experimental setup designed to evaluate the sound characteristics of the flexible electronic microphone system. To mimic the physiological features inside the neck where sound is generated during swallowing, we prepared a model consisting of a skin replica and an artificial esophagus. The white noise similar to that generated in a VFSS environment was played around the model, and a 200 Hz (a frequency range prevalent in swallowing sounds) chirp sound was transmitted through the artificial esophagus. We compared the filler materials under the microphone, such as soft gel or a rigid plate, to determine the ability to distinguish the swallowing sound beneath the skin replica while the external noise exists. The spectrograms and SNR levels depicted in Figure 3B–D collectively inform on the effectiveness of the gel in enhancing sound capture, particularly when conforming to curvatures. Specifically, using the gel maintains higher SNR levels on flat surfaces (Figure 3C) and preserves waveforms in the spectrogram that are absent without it (Figure 3D). The presence of second and third harmonic signals in Figure 3C, compared to their absence in Figure 3D, suggests that the gel helps maintain signal integrity by enhancing the contact between the device and the skin, which minimizes environmental noise. This effectiveness is attributed to the ability of the gel to adhere to the skin replica, even as the curvature increases, preventing noise infiltration and minimizing SNR reduction. On the other hand, the lack of gel results in decreased adhesion on curved surfaces, as evidenced by the significant gaps and increased noise in the spectrogram, highlighting the crucial role of the gel in consistent sound transmission as summarized in Figure 3B. This cohesive analysis clearly demonstrates the vital role of the gel in achieving optimal acoustic performance, ensuring fidelity in sound collection across varying surface topographies. In contrast, Figure 3D shows the signal processing without the encapsulating gel, which includes environmental noise and reduced signal clarity. The gel collects all harmonics and odd frequencies, which are sharper, fitting well to collecting the primary swallowing peaks while isolating the unwanted signals. To evaluate the sound‐capturing performance of our device during swallowing near the thyroid cartilage, we compared it with a commercial device, the ThinkLabs One (TLO) stethoscope. As shown in Figure 3E, our MEMS microphone‐based device with a gel layer underneath the fPCB securely conforms to the curvature of the thyroid cartilage, ensuring a minimal gap and optimal sound capture. The image and inset illustration highlight the seamless integration of the gel, fPCB, microphone, encapsulating silicone, and tacky tape on top. This combination emphasizes the ability of the device to adapt to the topography of the skin as well as minimize any motion artifacts from the gel layer conforming to the skin, and the tacky tape on top fully secures the whole microphone island. In contrast, Figure 3F depicts a commercial product, TLO, with a rigid plate that does not conform well to the thyroid cartilage, resulting in noticeable gaps that could potentially allow for the intrusion of ambient noise, thus affecting the sound capture capability of the device. The comparative image and inset underscore the lack of conformal contact between the device and the skin, suggesting a potential compromise in acoustic performance. A 2.5‐second window of a swallowing sound collected from our device is shown in Figure 3G and a side‐by‐side comparison by TLO is in Figure 3H. Figure 3G presents a spectrogram corresponding to sounds captured by our device, showcasing the temporal and frequency response of a swallowing event. The red blocks indicate moments of significant sound activity from the first, second, and third activation of EMG swallowing activities as well as corresponding to the swallow acoustic of interest against the background noise spectrum. Although our device captures sound during swallowing by presenting three clear peaks in corresponding time with swallowing EMG activity in Figure 3G, the TLO device generates distinct 500 Hz motion noise which may reflect the impact of the poor conformity of the device to the neck, and hence reducing the visibility of the three peaks in the spectrogram. This conclusive evidence validates our device's superior capability to accurately capture the critical sounds of swallowing, offering a promising alternative to traditional stethoscopes in both design and functionality.

### 2.4 Physiology of Swallowing and Detection of Abnormal Swallowing – Silent Aspiration by Using the Wearable System

**Figure 4:**
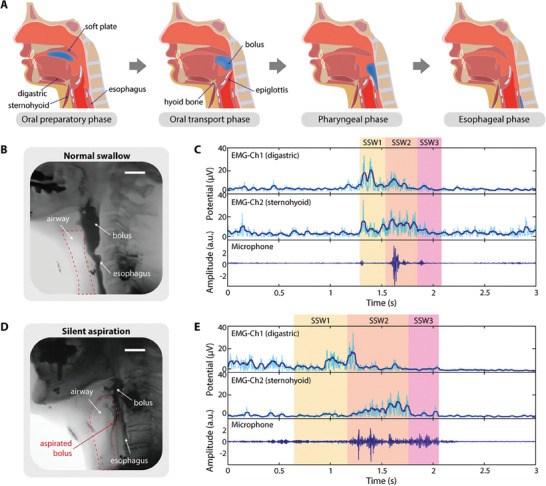
Physiology of swallowing and detection of abnormal swallowing – silent aspiration by using the wearable system. A) Schematic images showing swallowing phases. During the swallowing motion, the digastric and sternohyoid muscles, connected to the hyoid bone, repeat contraction and relaxation to transport the consistency from the mouth to the esophagus. B) X‐ray image of a normal swallow of 5 mL water, captured during VFSS. The airway is well protected while the bolus passes through the esophagus. Scale bar: 3 cm. C) Data measured by the wearable system with three sensors, showing a well‐synchronized sequence of muscle activities and sounds. D) X‐ray image of an abnormal swallowing of 5 mL water – silent aspiration. Since the airway is not closed well, the bolus penetrated or aspirated to the airway. Scale bar: 3 cm. E) Multimodal sensor data showing that when silent aspiration happens, muscle activities and swallowing sound are out of sync, leading to failure in closing the airway at the exact time.

The multimodal wearable swallowing monitor is designed to monitor the oropharyngeal swallowing process from the oral preparation stage to the esophageal stage.^[^ ^45^ ^]^ Specifically, this device monitors the sequential laryngeal complex movement by EMG signals and sound waves generated by swallowing which includes airway closure by the glottis and bolus pressurization and transit through a patent pharyngoesophageal segment to determine normal and dysphagic swallowing. We measured regions of the anterior digastric and sternohyoid muscles, which are connected to the hyoid bone located at the front of the neck and play a vital role in hyolaryngeal elevation and depression, which are involved in pharyngoesophageal segment opening and closing.^[^ ^46^ ^]^ Simultaneously, we measured three stages of swallowing sound waves (SSW) generated during the swallowing process, which are closely related to the movement of these muscles. The device monitored deglutition in all phases of swallowing, even during the provision of different consistencies of food such as liquids, soft food, and dense food (Figure S9, Supporting Information) where bolus preparation and swallowing differs (e.g., chewing, cough, and rest). In normal swallowing, each signal was measured in a well‐synchronized sequence, while it was not synchronized in the silent aspiration situation. Therefore, based on the sound wave and EMG patterns, sequence, and signal intensity and duration at this time, we were able to determine the normality of swallowing and detect differences between normal swallows and cases of silent aspiration. **Figure** **4**A illustrates the physiological processes involved in the four stages of swallowing, which are as follows: 1) Oral preparatory phase in Figure 4A: This is the initial stage of swallowing, which begins when the mouth opens, and the bolus enters the oral cavity and includes mastication and manipulation of the bolus. At this point, the entrance to the upper esophageal sphincter is contracted and the esophagus is closed, the epiglottis is in its native position, and the airway is open. 2) Oral transport phase in Figure 4A: The bolus is moved and positioned to the posterior aspect of the tongue in preparation for entry into the pharynx. 3) Pharyngeal phase in Figure 4A. The bolus moves through the pharynx into the esophagus, which is a complex sequence of events. The digastric muscle and other suprahyoid musculature activate, pulling and elevating the larynx toward the mandible. At this time, the vocal folds close to protect the airway, and the epiglottis retroflexes as the bolus is pressurized and propelled by the contraction of the base of the tongue and pharyngeal constrictors. Retroflexion of the epiglottis aids in guiding the bolus away from the glottis. The sound generated during this cohesive sequential process is the first swallowing sound wave (SSW1). The hyoid bone reaches its highest point, and then the counteracting force of sternohyoid and other infrahyoid musculature contraction depresses the hyoid bone down. This action closes the pharynx and the esophagus, creating the second swallowing sound wave (SSW2). 4) Esophageal phase in Figure 4A: The bolus moves through the esophagus into the stomach via rhythmic peristaltic smooth muscle contraction. At this time, the larynx has returned to its resting position. This elimination of the remaining bolus and restitution of the laryngeal position correlates with the third swallowing sound wave (SSW3) and marks the end of the oropharyngeal stages of swallowing. Normal swallowing progresses through well‐coordinated and sequential muscle movements as described. While swallowing, as shown in the X‐ray image (Figure 4B), the airway is well protected by epiglottis retroflexion and glottis closure while the bolus exits the pharynx and passes through the esophagus. These sequential movements can be observed by the synchronized EMG and sound signals measured by our device, as shown in Figure 4C. A group with a high incidence of dysphagia in stroke patients was recruited to investigate abnormal swallowing. As shown in Figure 4D, when silent aspiration occurred, the bolus transition time was longer than normal swallowing, and the activation of EMG showed a different pattern as well as a less organized sequence of swallowing sounds. This shows a less harmonious activation of the swallowing apparatus as well as a failed response to the aspirated material due to the underlying neural injury. A distinctive pattern between normal swallowing and silent aspiration would provide reliable data to determine silent aspiration using our device's deep learning algorithm.

### 2.5 Development of a Deep‐Learning Algorithm for Automated Classification of Swallowing Status and Detection of Abnormality with Multiple Post‐Stroke Patients

**Figure 5:**
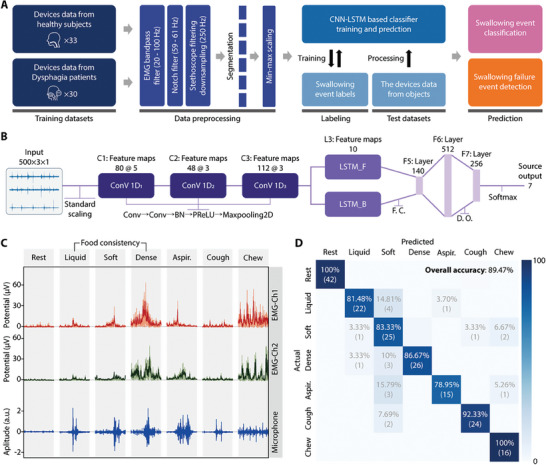
Development of a deep‐learning algorithm for automated classification of swallowing status and detection of abnormality with multiple post‐stroke patients. A) Schematic overview of the data processing and machine learning for swallowing status classification and silent aspiration detection. B) Machine learning architectures for data classification; Conv: Convolution, FC: Fully Connected layers, DO: Dropout, and BN: Batch Normalization. C) Measured swallowing data from the multimodal wearable system during seven classes, including rest, liquid, soft food, dense food, silent aspiration, cough, and chewing. D) Confusion matrix showing the model classification test accuracy of 89.47% of seven classes for swallowing status classification.

The current gold standard for analyzing swallowing patterns and detecting dysphagia is the VFSS, which requires an X‐ray imaging instrument, and highly trained personnel to observe visual data and manually score radiographic images. This method causes delays, additional costs, and human errors and also restricts monitoring to specific hospital settings.^[^ ^47^ ^]^ More importantly, swallow monitoring with the VFSS has low accessibility, which results in extended periods between monitoring sessions for patients, creating gaps in monitoring. To address these challenges, we developed an automated swallowing status classification and swallowing dysfunction detection method using a hybrid deep learning structure, utilizing CNN‐LSTM hybrid algorithm. This model combines the spatial feature extraction capabilities of CNNs with the sequential data interpretation strengths of LSTMs, offering enhanced accuracy and dynamic analysis in real‐time.^[^ ^48^ ^]^ Notably, similar hybrid deep learning approaches have been extensively applied in swallowing physiology fields,^[^ ^49^, ^50^, ^51^, ^52^, ^53^ ^]^ demonstrating effectiveness in capturing complex patterns and time‐dependent structures in diverse datasets. **Figure** **5**A illustrates the overall progression from data processing to training, processing, and prediction. Swallowing data from 33 healthy participants and 30 dysphagia patients (Tables S1 and S2, Supporting Information) were used to train the CNN‐LSTM‐based swallowing status classification. The CNN‐LSTM hybrid model proposed in this work receives epoch‐by‐epoch 2‐s‐long filtered EMG and sound signals as inputs, which are then directed to the CNN‐LSTM hybrid model after data preprocessing. Figure 5B presents the CNN‐LSTM hybrid model architecture, utilizing swallowing data acquired from the wearable swallowing patch for automated swallowing assessment. To begin, we collected EMG and sound data that were measured concurrently with the VFSS exam and accurately labeled by medical professionals. In order to expand our dataset and address the limited availability of silent aspiration samples, we applied a data augmentation technique, which involved shifting the 2‐s epochs by 0.2 s in the positive direction. Each shifted sample was then carefully re‐labeled manually, ensuring the data's accuracy. The labeled data was organized into a single dataset with distinct folders for each class and underwent preprocessing, including one‐hot encoding for the class labels. Following this, we employed an interpatient split strategy to divide the dataset into training, validation, and testing sets in a 60:20:20 ratio, while adjusting to ensure that augmented data from individual participants did not mix between these sets. For example, if data from Participant 1 was included in the training set, it was not allowed to appear in the validation or test sets. This model features three convolutional blocks, each complemented by two one‐dimensional Convolutional layers (Conv 1D), Batch Normalization (BN), and Parametric Rectified Linear Units (PReLU) activation. Following these, we integrated a bidirectional LSTM layer (tanh activation) and three dense layers (PReLU activation), incorporating dropout to prevent overfitting. The architecture culminates in a softmax activation layer, classifying the data into seven distinct categories: rest, liquid, soft food, dense food, silent aspiration, cough, and chewing based on their different signal features, as depicted in Figure 5C. More detailed information about the layers and parameters of our deep learning model can be found in the Experimental Section and Table S3 (Supporting Information). The confusion matrix presented in Figure 5D depicts the comparative performance of our CNN algorithm against manual scoring using VFSS. The algorithm achieved an overall accuracy of 89.47%, demonstrating a close alignment with traditional VFSS manual scoring (**Table** **1**). Specifically, the sensitivity and specificity for each class are as follows: for resting periods, both sensitivity and specificity are 1.00; for liquid, the sensitivity is 0.81 and the specificity is 0.99; for soft foods, the sensitivity is 0.83 and the specificity is 0.93; for dense foods, the sensitivity is 0.87 and the specificity is 0.99; for aspiration events, the sensitivity is 0.79 and the specificity is 0.99; for cough, the sensitivity is 1.00 and the specificity is 0.98; and for chewing movements, the sensitivity is 0.92 and the specificity is 0.99. These results affirm the reliability of our algorithm in monitoring swallowing status and dysfunction, which is further supported by the automatic reports generated from each session (Video S2, Supporting Information).

**Table 1:** Comparison of wearable monitoring systems for swallowing detection.

References	Motion artifact control	Measured signal	Sensor type	Sensor location	Classification method	Accuracy (number of classes)	# of subjects (normal/patient)	Verification via VFSS	Food consistency variation	Aspiration detection
This work	Yes	EMG and sound	Soft dry electrode/MEMS microphone	Chin, neck	Machine Learning	89.47% (n = 7)	33/30	Yes	Yes	Yes
[21]	–	EMG	Commercial Ag/AgCl electrodes	Chin, neck, cheek, mouth	–	–	15/14	–	–	–
[22]	–	EMG, strain	Soft electrode/piezoresistive strip‐based strain gauge	Chin, neck/laryngeal	–	–	4/1	Yes	–	–
[26]	–	Acceleration	IMU^*^	Throat, ribcage	Machine Learning	90% (n = 3)	184/321	–	–	–
[28]	–	Pressure	Single‐walled carbon nanotube‐based pressure sensor	Neck	Algorithm	68.5–96% (n = 4)	10/0	–	–	–
[27]	–	Acceleration	IMU	Neck, upper chest	Algorithm	91% (n = 2)	67/4	–	–	–
[17]	–	Sound	Microphone	Neck	Algorithm	88.8% (n = 2)	8/62	–	–	–
[23]	–	EMG	Soft electrode	Submental	–	–	4/0	–	–	–
[29]	–	Strain	AIN^**^ Piezoelectric	Neck	–	–	8/0	–	–	–
[30]	–	Sound, acceleration	High‐resolution cervical auscultation	Neck	Machine Learning	82.84% (n = 2)	0/189	Yes	–	Yes
[31]	–	Sound	iPad microphone	Neck	Machine Learning	86.6% (n = 2)	0/449	–	–	Partially
[32]	–	Sound	Electret condenser microphone	Neck	Machine Learning	88.75% (n = 4)	–	–	Yes	–
[33]	–	Sound	Acoustic microphone	Neck	Machine Learning	84.9% (n = 4)	12/0	–	Yes	–

* IMU: Inertial Measurement Unit

** AIN: Aluminum Nitride

## 3 Conclusion

This paper presents a wireless neck‐wearable system for automatic clinical assessment of swallowing behavior and diagnosis of silent aspiration. Unlike existing wearable monitoring devices, the multi‐functional sensor system presented in this work can detect episodic silent aspiration occurring in a patient with dysphagia via continuous, real‐time, portable swallowing monitoring. Our wearable and portable system provides a means to utilize non‐invasive surface multimodal monitoring of deglutition and represents the first report of direct detection of both silent aspiration symptoms and the food types via a wearable device without relying on VFSS. This wearable platform seamlessly bridges the discontinuous boundaries between humans and machines through the effective processing of soft materials, offering stable and consistent multimodal monitoring. Through the machine learning model built using the collected data, the wearable system detects overall swallowing status, including rest, food consistency, silent aspiration, cough, and chewing, with high accuracy. This device enables at‐home monitoring of swallows. Therefore, this device significantly enhances the accessibility of swallowing monitoring for patients and can fill the monitoring gap that occurs between the first VFSS and the subsequent hospital visit for another VFSS. This allows patients, such as stroke survivors, to reduce the frequency of their hospital visits while still receiving more continuous monitoring, thereby reducing potential risks from food intake and safely undergoing rehabilitation treatment with less dependence on specialized facilities. Our future research will integrate additional sensing functions and target large‐scale clinical studies of dysphagia patients with various underlying diseases.

## 4 Experimental Section

### Device Circuitry Design

A variety of electronic components were incorporated into the design of the soft wearable sensor for monitoring swallowing dysfunctions. Alongside the standard passive elements like resistors, capacitors, and inductors, the circuitry also integrated the BLE SoC (nRF52832, Nordic Semiconductor). Two ADS1292 chips from Texas Instruments were crucial: one dedicated to capturing signals from EMG channels 1 and 2, and the other specifically for processing the microphone signal. The design further incorporated the ICS40212 MEMS microphone chip from TDK InvenSense and the TS472 audio amplifier chip sourced from ST Electronics and concluded with the TPS63001 switching voltage regulator chip, ensuring a consistent voltage supply across the circuit.

### Device Fabrication

The construction of the suggested system encompassed three primary phases: fabricating the fabric base, circuit fabrication and encapsulation, and developing the EMG nanomembrane electrode, followed by the final assembly. The process of fabric base creation began by combining Silbione parts A and B in equal weight proportions, spinning this mixture on a PTFE sheet at 1200 RPM, then applying brown medical tape and baking at 65 °C. Once set, the PTFE sheet was removed. In the study, the fabrication yield for the kirigami electrodes was 95%, and for the entire encapsulated circuitry, it was 90%, based on the production of over 20 devices. Losses occurred primarily during the peeling process after laser micromachining and due to shorting of some chip components during soldering. The power component consisted of a lithium polymer battery, a slide switch, and a magnetic charging dock. To shield the integrated circuit from strain, a low‐modulus elastomer was placed beneath it, and an additional elastomer encased the whole electronic setup, leaving only the switch and charging port visible. The EMG nanomembrane electrode creation process involved gold/chromium electrodes, applied via E‐beam evaporation and simple laser cutting. PDMS was chosen as the foundational layer for the electrode because of its adhesive properties and ease of detachment. A polymer film was attached to the PDMS, followed by the deposition of gold, and the film underwent laser ablation to create a flexible electrode design. Unnecessary materials were manually removed from the PDMS. In the final assembly, gold electrodes were shifted onto the adhesive side of the fabric using water‐solvent tape. After laser patterning the electrode‐embedded fabric, the encapsulated electronic system was connected to the side of the fabric by adding and curing a silicone layer. This device demonstrated less than 10% performance degradation even after more than 20 repeated sessions, confirming its suitability for repeated use.

### Characterization of Mechanical Properties of the Device

The testing setup for both mechanical and electrical assessment included utilizing a digital force gauge (M5‐5, Mark‐10) in conjunction with a motorized testing platform (ESM303, Mark‐10) for evaluating mechanical properties, as well as an LCR meter (Model 891, BK Precision) to quantify electrical resistance. To evaluate cyclic stretching, the electrode system underwent alternating stretching and releases in a vertical direction at a rate of 25 mm min^−1^, repeated for 300 cycles. In contrast, this stretching and releasing were conducted for 300 cycles at the same rate for the interconnector strain assessment. To evaluate the impedance of electrodes when in contact with the skin, the gold electrode was transferred onto adhesive brown tape. A comparative test was conducted using a gel electrode to analyze the impedance. This assessment was done on the chin after cleaning the electrode placement site with NuPrep Skin Prep Gel from Weaver & Co. An impedance meter (1089NP Checktrode, UFI) was used in tandem with two skin‐based electrodes to measure this contact impedance. For the SNR computation of the data, the inactivity period prior to swallowing was considered noise. The amplitude of this noise was then determined. The SNR value was calculated using the following equation: SNR_dB_ = 20log_10_(A_Signal_/A_Noise_).

### FEA Modeling

3D FEA was utilized to determine the mechanical deformations and strain distributions when the system was applied to human skin. The simulation incorporated eight‐node 3D solid elements for the fabric and Silbione, and a two‐layer structure (gold/PI for the electrode and copper/PI for the connector) using four‐node shell elements for the kirigami patterned EMG electrodes. The simulation mesh was optimized to guarantee computational accuracy. The side surfaces of the fabric were assigned displacement type boundary conditions, where different levels of stretch were exerted. The threshold for elastic stretchability was marked by the metal layer undergoing strain beyond its yield strain across a minimum of half the width of any section. The simulations considered the material properties, such as the Young's modulus (E) and Poisson's ratio (ν), with values being: *E*_au_ = 79 GPa and ν_au_ = 0.44 for gold, *E*_pi_ = 2.5 GPa and *ν*_pi_ = 0.34 for polyimide, *E*_cu_ = 118 GPa and *ν*_cu_ = 0.34 for copper, and values for the kirigami patterned fabric and Silbione as *E*_f_ = 1.28 MPa, *ν*_f_ = 0.184 and *E*_si_ = 5 kPa, *ν*_si_ = 0.48 respectively. The optimized mesh size was identified through the mesh convergence test and applied it to the FEA. When the global mesh size was 0.1, further refinement had less than a 1% impact on the maximum strain, allowing for accurate simulation without excessive computation.

### Protocol for Collecting Swallowing Data

Prior to the swallowing test protocol, subjects had a preliminary EMG pre‐test application (Figure S10, Supporting Information) to ensure that the EMG electrode was aligned with the direction of the target muscle fibers to measure the EMG signals accurately. The procedure began with the subjects performing two swallows of water, separated by a 5‐s interval, to facilitate the exportation and filtering of the raw EMG data alongside the corresponding SNR values. Locations that yielded SNR values above 6 dB were deemed optimal for placing the EMG electrodes. Once these locations were established, subjects proceeded to the actual swallowing test protocol. In the swallowing test protocol utilizing the soft patch device, subjects underwent evaluation of their swallowing capabilities with various consistencies, including water, thickened water, apple sauce, pudding, fruit cup, and crackers, all mixed with barium sulfate for contrast visualization for videofluoroscopy. The accompanying mobile application of the multimodal wearable swallowing monitor was initiated with the “Start Recording” feature to display real‐time graph results. As subjects consumed each food type, the “Bolus Preparation” button was tapped before swallowing, followed by the “Swallow” button for precise timing. A short “Pause” option was available as needed. Once all test items were consumed, “End Recording” was activated to finalize data, ensuring comprehensive assessment and analysis of each swallow event (Video S1, Supporting Information).

### Data Processing Method

To ensure optimal portability and functionality of the wearable system, given the computational demands, the signal capture and processing framework was split across three distinct electronic platforms: i) wearable swallowing monitor, ii) mobile devices like smartphones or tablets, and iii) a personal computer (PC). Initially, the wearable system detects signals through sensors positioned on the skin and transmits them to the mobile device for storage through Bluetooth. Subsequently, this collected data was transmitted to a PC, where it undergoes complex signal processing and CNN‐LSTM based analysis, requiring substantial computational resources. All data processing was executed in Python. More specifically, the EMG data collected from the setup was subjected to bandpass filtering (ranging from 20 to 100 Hz) and notch filtering (spanning 59–61 Hz) to eliminate various interference, including drifts, electrical disturbances, ambient noise, and others. In processing the sound signals acquired from the MEMS microphone, a multi‐stage procedure was employed to ensure clarity and accuracy. First, raw sound data were digitized at a sampling rate of 2 kHz, a standard rate that offers a broad frequency range from 0 to 1 kHz, particularly crucial for capturing the nuances in swallowing sounds. Noise reduction algorithms were then applied to eliminate potential artifacts or background noises. The sound signals were subsequently passed through a band‐pass filter to isolate the frequencies of interest within the 10–900 Hz range. Fourier transform was applied to transition from the time domain to the frequency domain, facilitating the identification of specific frequency components and patterns related to swallowing or other physiological activities. For temporal analysis and event detection, the processed signals were further analyzed using a short‐time Fourier transform technique, enabling the observation of changes in frequency components over time.

### Classification of Swallowing

In the research on swallowing patterns using EMG and sound signals, a deep learning architecture was employed that integrates both CNN and LSTM layers. This model was constructed using TensorFlow 2.0 in Python and executed on a laptop equipped with an Intel i7 processor (I7‐9750H). The EMG and sound signals (250 Hz), segmented into 2‐s intervals, underwent a normalization process to have values ranging between 0 and 1 using the Standard Scaler method. This study strategically divided the dataset: 60% was allocated for training (570 samples), 20% for validation (190 samples), and the remaining 20% for testing (190 samples). The architecture begins with an input layer for EMG signals, followed by three convolutional blocks. Each block has two convolutional layers: the first layer varies in filters and kernel sizes (80 filters with kernel size 5, 48 filters with kernel size 3, and 112 filters with kernel size 3, respectively), while the second layer in each block uses 16 filters with a kernel size of 4. Each block includes PReLU activation and max‐pooling. The architecture features a bidirectional LSTM layer with ten units and tanh activation, followed by a sequence of dense layers featuring PReLU activation: the first consists of 140 units, the second contains 512 units with a 0.2 dropout for regularization, the third comprises 256 units, and the final output layer uses softmax activation to categorize the outputs. For the optimization process, the ADAM optimizer with a fixed learning rate of 0.001 was utilized, and losses were computed using the categorical cross‐entropy function. Throughout the training, the weights of the model were dynamically adjusted based on validation accuracy, and an array of hyperparameters was selected through Keras Tuner's random search method. The ultimate model, showing the highest validation accuracy, was then put to the test to gauge its predictive ability. More detailed information about the learning curve and hyperparameter tuning of the deep learning model can be found in Figure S11 and Table S4 (Supporting Information).

### Human Subject Study

The research included both healthy participants and individuals with symptoms. Healthy volunteers, aged between 18 and 40, participated in the study under the approved IRB protocol (#H21038) at Georgia Tech, and IRB protocol (STUDY00003895) at Emory University. Meanwhile, patient‐based research was carried out at Chungnam National University Hospital (CNUH) in line with IRB protocol (#CNUH 2022‐12‐073). The CNUH team received the device components and finalized the investigational device for the study. Prior to enrollment, all participants were extensively briefed on the study procedures and provided informed consent. Participants with dysphagia, aged 19 to 90 years, were included if they or their guardians could understand and consent to the study and had a BMI less than 30 to facilitate accurate EMG measurements. Healthy controls, also aged 19 to 90 years, were included if they did not exhibit symptoms of dysphagia and could independently consent to participate. Exclusion criteria encompassed participants or guardians unable to provide consent, individuals below 19 or above 90 years of age, and those with a BMI of 30 or higher, as excessive adiposity in the neck area could affect EMG signal accuracy. Detailed demographic and clinical characteristics of both groups are summarized in Tables S1 and S2 (Supporting Information), respectively.

## Conflict of Interest

Georgia Tech has a pending patent application based on the materials described here.

## Author Contributions

B.S., S.H.L., and K.K. contributed equally to this work. B.S., M.‐K.Y., H.C., and W.‐H.Y. designed the research. B.S., S.H.L., K.K., Y.L., N.C., S.A., S.S., N.S., and A.T. performed the research. B.S., S.H.L., K.K., Y.L., A.T., M.‐K.Y., H.C., and W.‐H.Y. analyzed the data. B.S., S.H.L., K.K., M.‐K.Y., H.C., and W.‐H.Y. wrote the paper.

## Supporting information

Supporting Information

Supplemental Video 1

Supplemental Video 2

## Data Availability

The data that support the findings of this study are available from the corresponding author upon reasonable request.
