# Supplementary material for: Automatic Clinical Assessment of Swallowing Behavior and Diagnosis of Silent Aspiration Using Wireless Multimodal Wearable Electronics
Source: Adv Sci (Weinh). 2024 Jul 9;11(34):2404211. doi: 10.1002/advs.202404211 (PMC11425633; doi:10.1002/advs.202404211)
Supplement: Supplementary file 1 — Supporting Information [file ADVS-11-2404211-s003.pdf]

ADVANCED SCIENCE
Open Access

Supporting Information

for *Adv. Sci.*, DOI 10.1002/advs.202404211

Automatic Clinical Assessment of Swallowing Behavior and Diagnosis of Silent Aspiration Using Wireless Multimodal Wearable Electronics

Beomjune Shin, Sung Hoon Lee, Kangkyu Kwon, Yoon Jae Lee, Nikita Crispe, So-Young Ahn, Sandeep Shelly, Nathaniel Sundholm, Andrew Tkaczuk, Min-Kyung Yeo, Hyojung J. Choo* and Woon-Hong Yeo**

Supporting Information

Automatic clinical assessment of swallowing behavior and diagnosis of silent aspiration using wireless multimodal wearable electronics

Beomjune Shin†, Sung Hoon Lee†, Kangkyu Kwon†, Yoon Jae Lee, Nikita Crispe, So-Young Ahn, Sandeep Shelly, Nathaniel Sundholm, Andrew Tkaczuk, Min-Kyung Yeo, Hyojung J. Choo* and Woon-Hong Yeo**

B. Shin†, S. H. Lee†, and K. Kwon†
Equally contributed to this work.

B. Shin, W.-H. Yeo*
George W. Woodruff School of Mechanical Engineering, Georgia Institute of Technology, Atlanta, GA 30332, USA
*E-mail: whyeo@gatech.edu

B. Shin, S. H. Lee, K. Kwon, Y. J. Lee, N. Crispe, W.-H. Yeo
Wearable Intelligent Systems and Healthcare Center (WISH Center), Institute for Matter and Systems, Georgia Institute of Technology, Atlanta, GA 30332, USA

S. H. Lee, K. Kwon, Y. J. Lee
School of Electrical and Computer Engineering, Georgia Institute of Technology, Atlanta, GA 30332, USA

N. Crispe, W.-H. Yeo
Wallace H. Coulter Department of Biomedical Engineering, Georgia Institute of Technology and Emory University School of Medicine, Atlanta, GA 30332, USA

S.-Y. Ahn
Department of Rehabilitation Medicine, Chungnam National University School of Medicine, Daejeon 35015, Republic of Korea

S. Shelly, N. Sundholm, A. Tkaczuk
Department of Otolaryngology - Head and Neck Surgery, School of Medicine, Emory University, Atlanta, GA 30322, USA

M.-K. Yeo*
Department of Pathology, Chungnam National University School of Medicine, Daejeon 35015, Republic of Korea
*E-mail: mkyeo83@cnu.ac.kr

H. J. Choo*
Department of Cell Biology, School of Medicine, Emory University, Atlanta, GA 30322, USA
*E-mail: hyojung.choo@emory.edu

W.-H. Yeo*
Parker H. Petit Institute for Bioengineering and Biosciences, Institute for Robotics and Intelligent Machines, Georgia Institute of Technology, Atlanta, GA 30332, USA

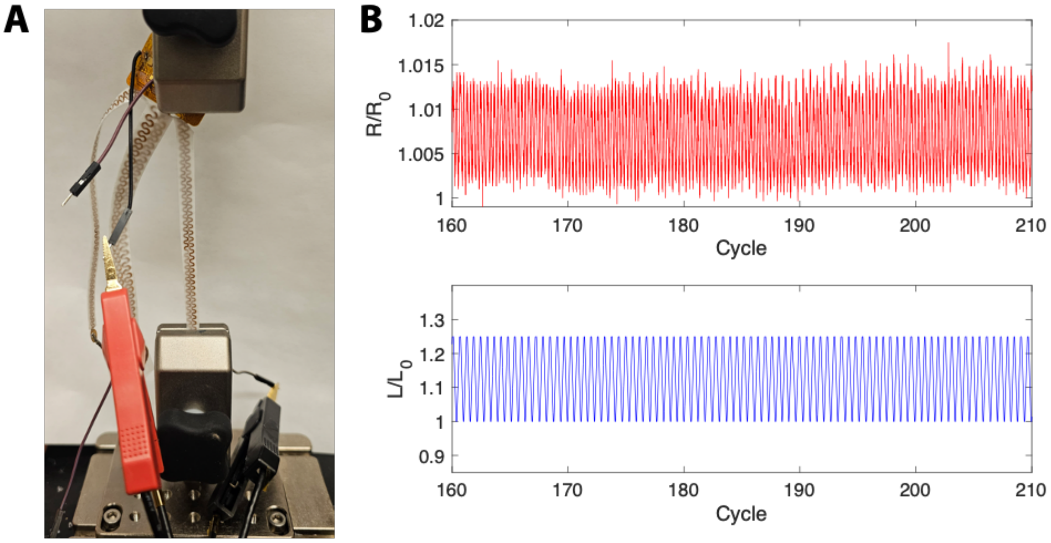

Figure S1. Interconnector strain test. (A) Test setup for the multimodal wearable device on Mark-10 to test the strain of the interconnects. (B) The hardware-level behavior of the entire device shows resistance change over 210 cycles.

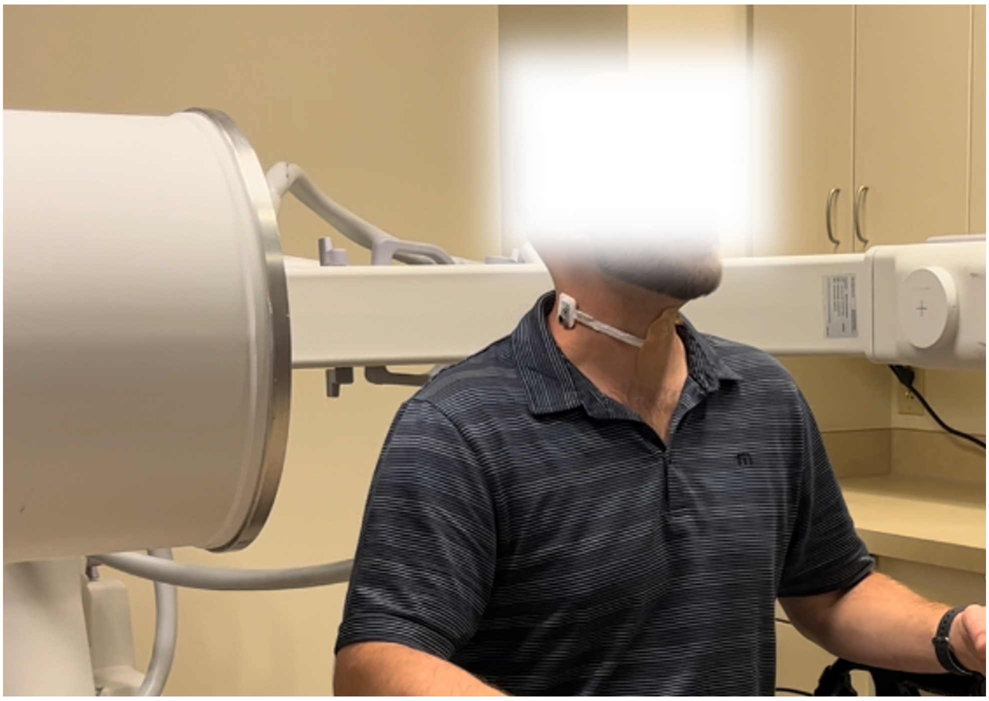

Figure S2. VFSS exam setup with the multimodal wearable device.

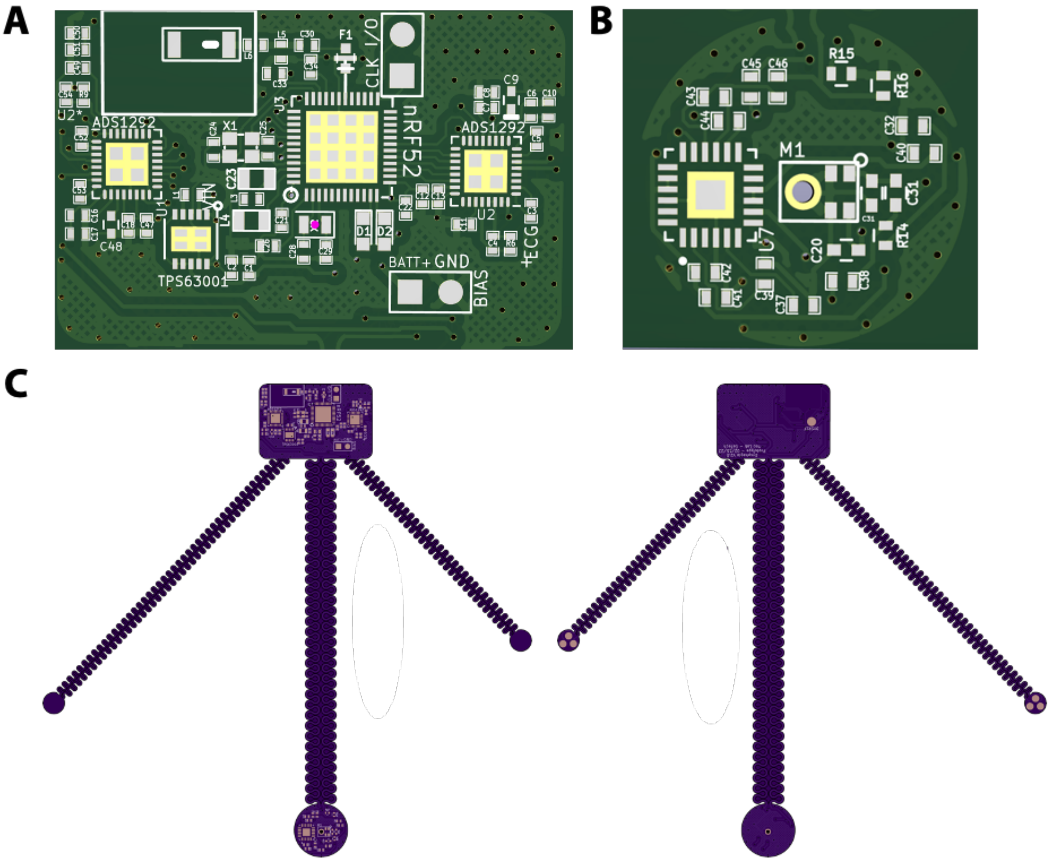

Figure S3. Multimodal wearable device circuit. (A) Mainboard with nRF, one ADC for a microphone, and one ADC for two-channel EMG (top view). (B) Microphone island with MEMS microphone and the preamplifier (top view). (C) The entire device, front and back, has EMG electrode pads on the bottom layer.

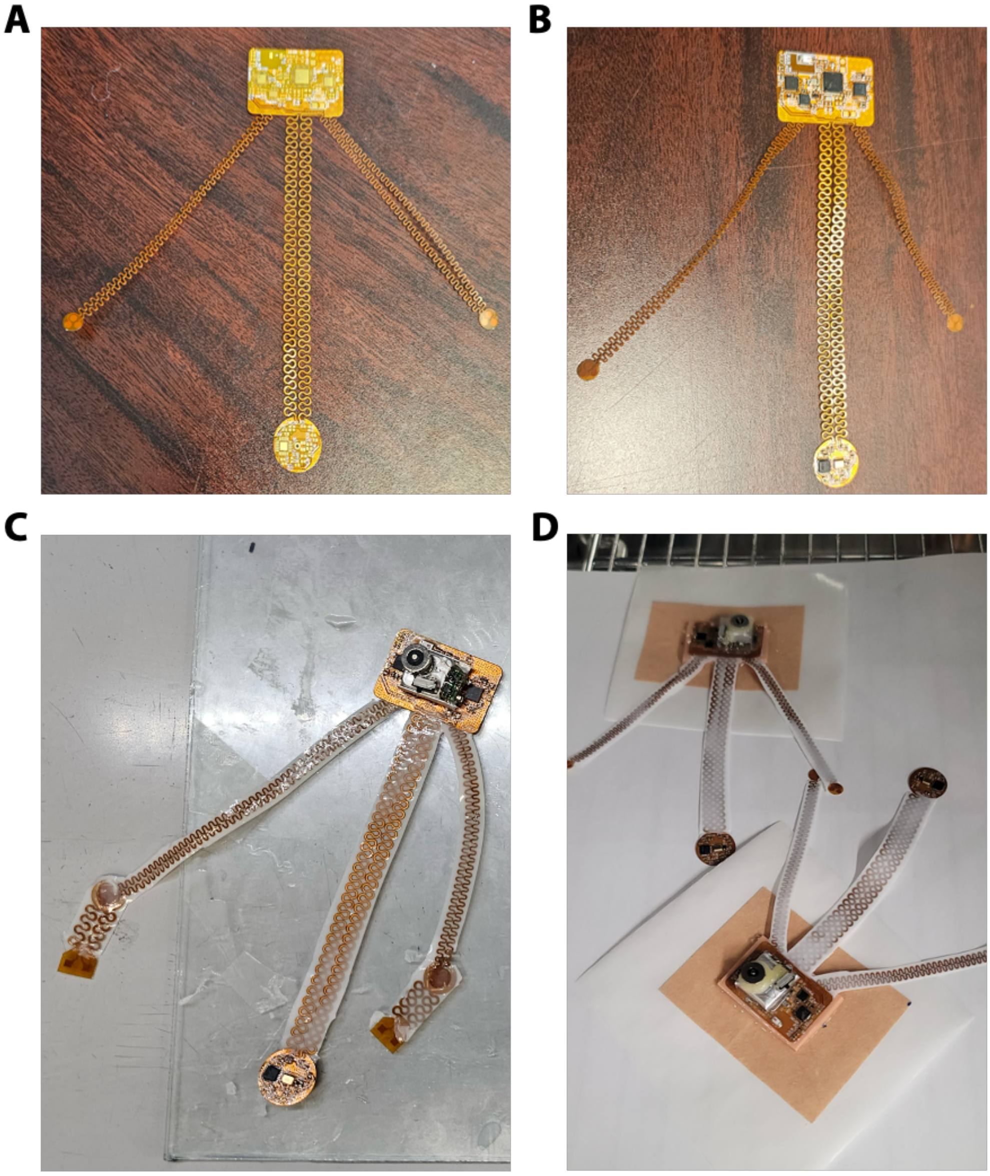

Figure S4. Multimodal swallowing monitor fabrication process. (A) Bare fPCB board with EMG electrode joints. (B) Reflow soldered chips are integrated into the fPCB with a firmware-programmed microcontroller. (C) EMG joints are soldered to the fPCB and the battery to power up the entire circuit. (D) EMG reference electrode with the silbione layer integrated on the bottom of the main island along with encapsulation for the whole device.

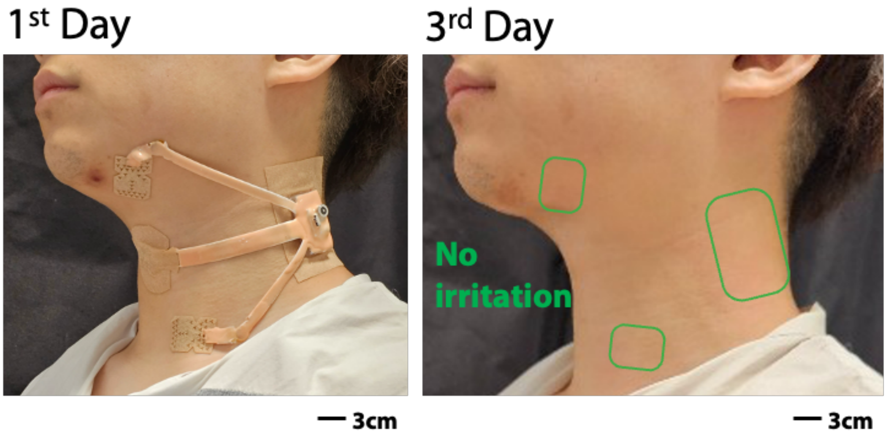

Figure S5. Long-term skin compatibility testing. The entire device has been worn for three days, and there has been no skin irritation along the parts directly touching the skin.

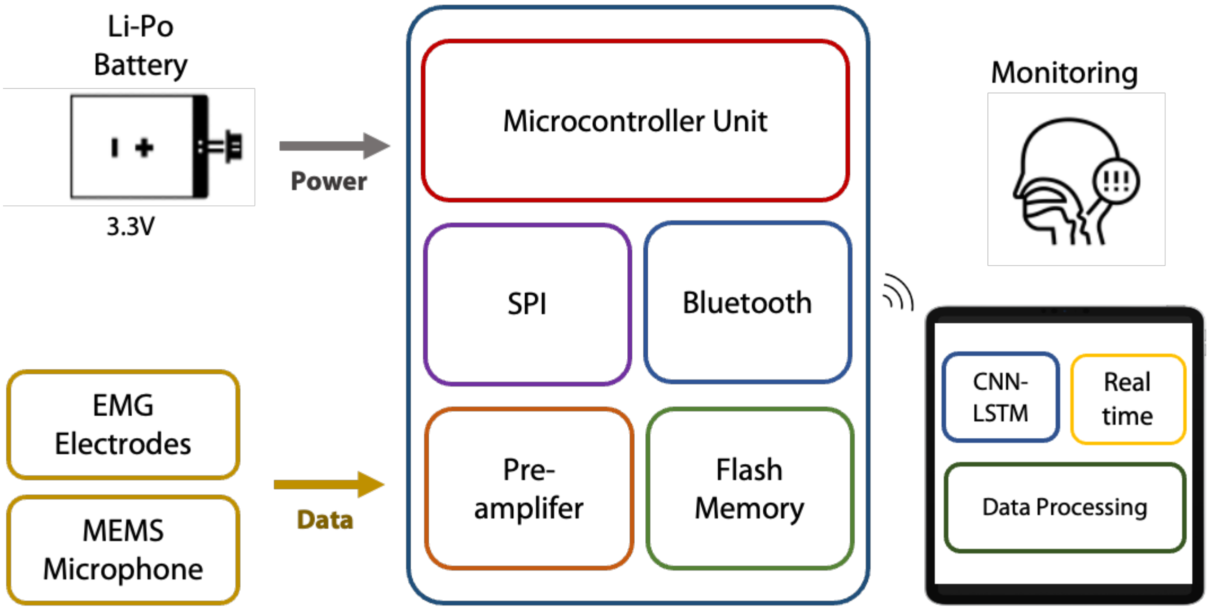

Figure S6. Schematic illustration of the device on power, data, hardware structure, and the real-time security system.

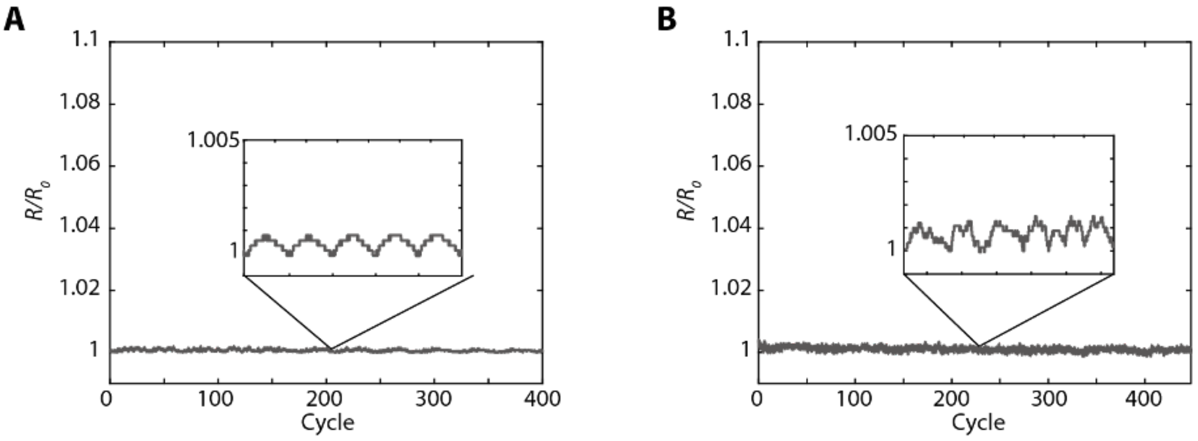

Figure S7. The resistance change of electrodes attached to the patch with various kirigami patterns. The resistance was measured by repeating 25% strain by Mark-10. (A) The result of the electrode with a t-shape kirigami patterned patch and (B) non-kirigami patterned patch.

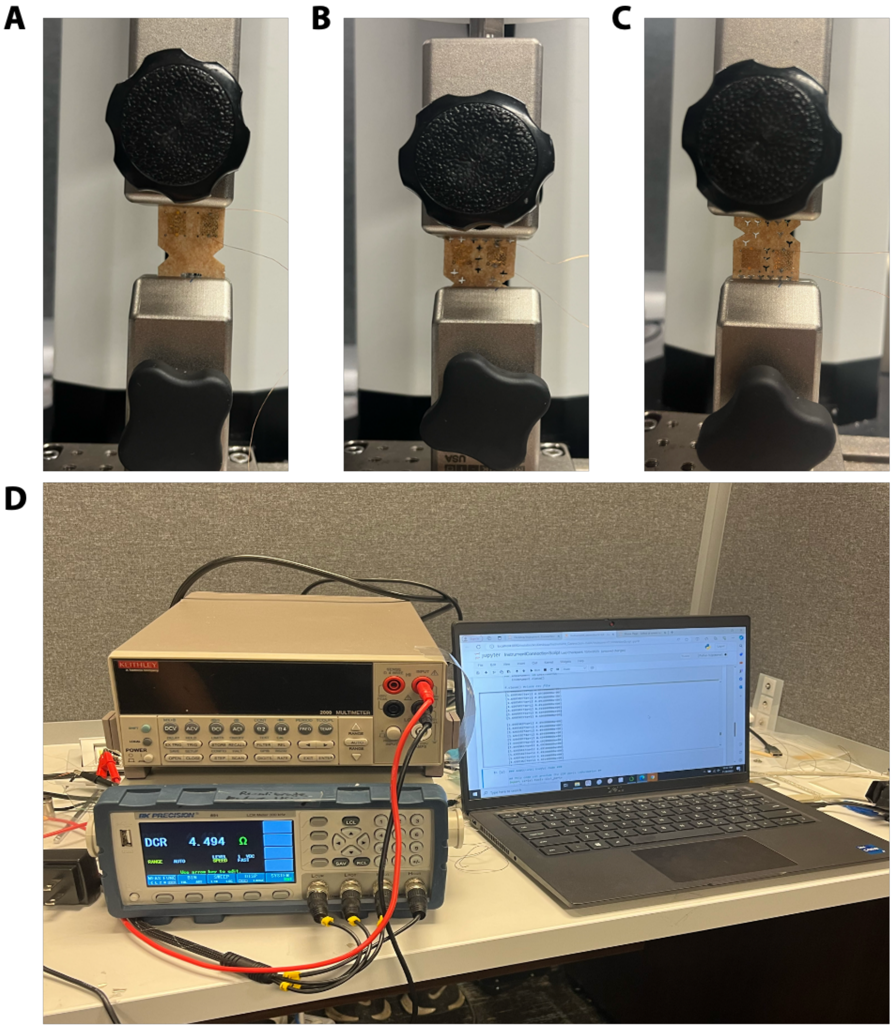

Figure S8. Mechanical test setup for the EMG electrodes. (A) EMG electrode without kirigami pattern under Mark-10. (B) EMG electrode with t-shaped kirigami pattern. (C) EMG electrode with y-shaped kirigami pattern. (D) The entire setup of the data acquisition system for Mark-10 with an LCR meter connected to a Python program to continuously read BK Precision values.

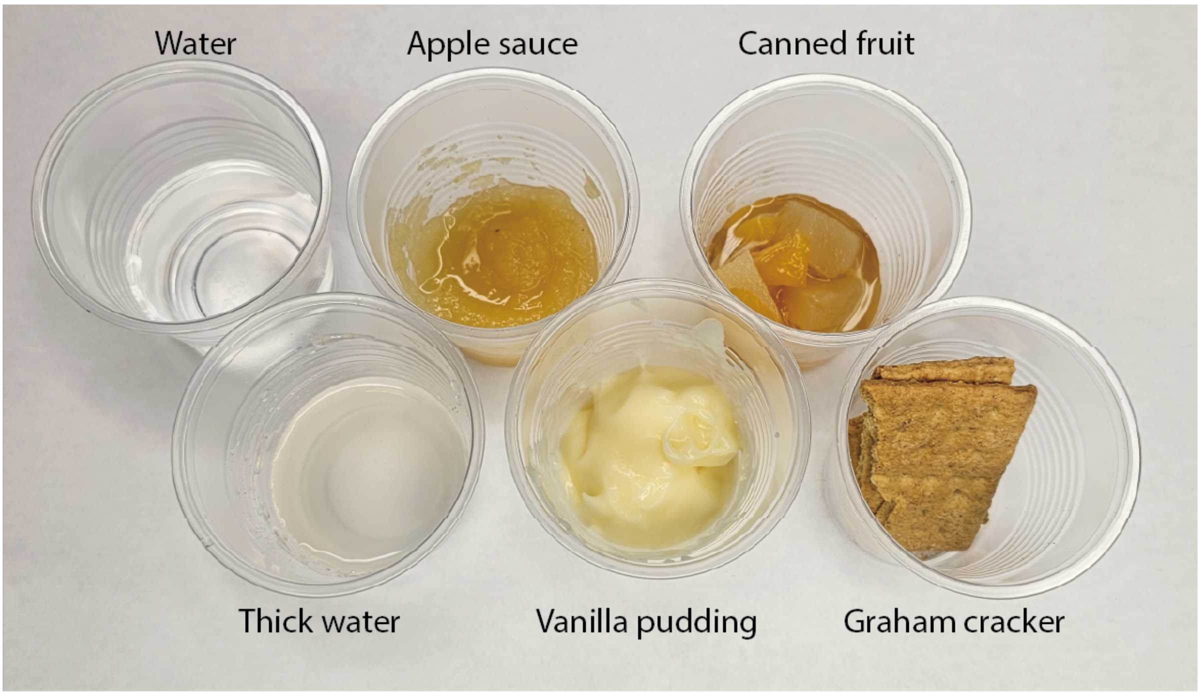

Figure S9. Foods used for the test have various consistencies. According to the consistency of each sample, six types of foods, water, thick water, apple sauce, vanilla pudding, canned fruit, and graham crackers, are categorized into three groups: liquid, soft food, and dense food. Each group includes two food consistencies in order from the front of the listed foods. When conducting the VFSS exam, barium sulfate was added to each type of food to make them observable through videofluoroscopy.

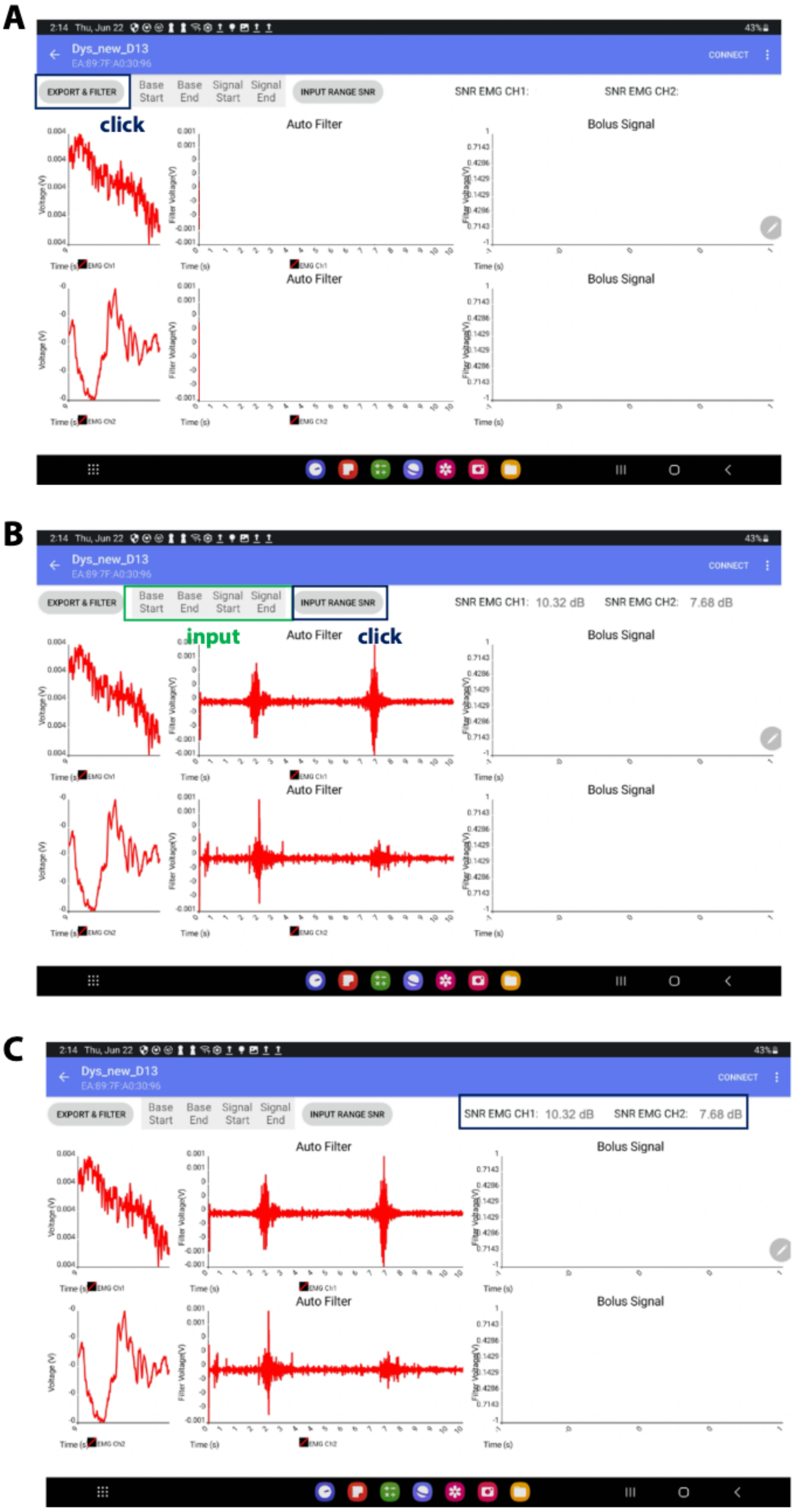

Figure S10. EMG electrode pre-test application. EMG test app with two swallows with a 5-second delay between two swallows. (A) Real-time raw EMG data for the top and bottom electrodes are displayed on the left during the swallow monitoring. Then, the export and filter button is pressed. (B) The filtered data is shown in the middle. Enter the time for the peak and base areas and press the input range SNR button. (C) The SNR of the signals collected from each electrode is displayed.

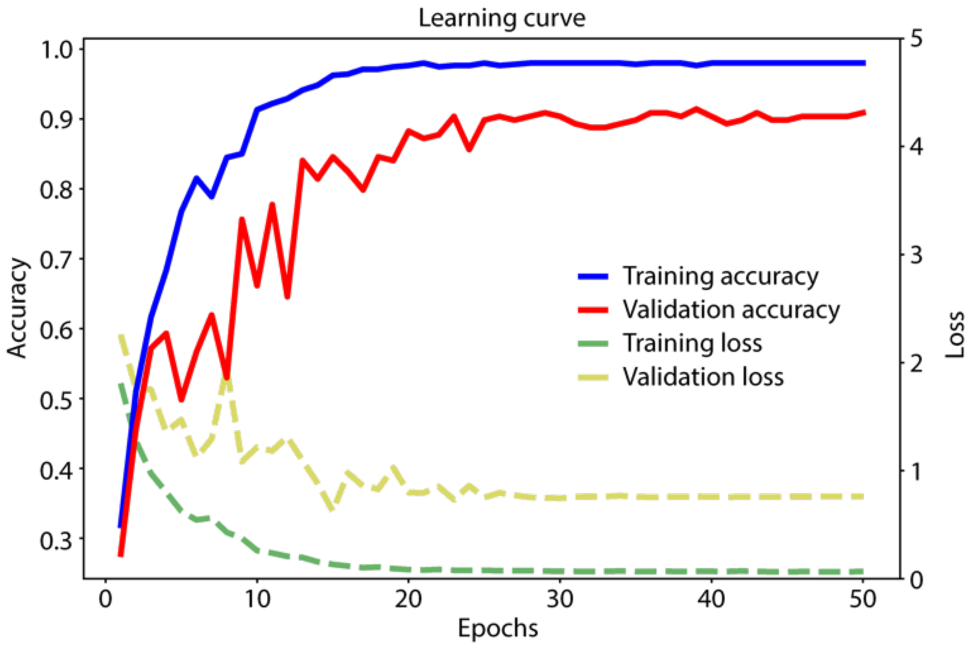

Figure S11. Learning curves of the training on the swallowing status classification mode.

Table S1. Information on health controls.

Subject Number	Gender, Age	BMI	Subject Number	Gender, Age	BMI
1	F, 30	22.3	18	M, 51	29
2	M, 24	20.1	19	F. 42	23.2
3	F, 22	17.4	20	F, 42	34.4
4	M, 24	22.6	21	M, 61	25.9
5	F, 22	24.2	22	F, 57	34.1
6	F, 19	20.7	23	F, 69	30.7
7	F, 49	26.2	24	F, 67	16.8
8	F, 23	22.2	25	F, 63	30.4
9	F, 20	21.8	26	F, 28	22.5
10	F, 40	19.3	27	F, 32	21.1
11	F, 44	23.6	28	M, 43	22.4
12	M, 41	21.4	29	F, 28	19.5
13	F, 32	18.7	30	F, 63	18
14	F, 48	28.2	31	F, 25	23.9
15	F, 47	23.5	32	M, 34	18.9
16	M, 58	22.9	33	F, 60	54.1
17	M, 58	25.2			

Table S2. Information of participants with dysphagic symptoms.

Patient	Gender, Age	BMI	Underlying Disease	Swallowing Reflex	Soft1	Soft2	Water 2.5mL	Water 5mL	Dense	ASHA NOMS Level
Aspiration Patients										
1	F, 80	18.7	S-ICH(12/28/22), HTN	Delayed	2	1	2	2	N/A	5
2	F, 82	20.5	CI(07/17/23), DM, HTN, CKD	Intact	1	1	1	1	N/A	5
3	M, 74	22.2	CI(06/05/23), DM, HTN	Delayed	1	1	7	N/A	N/A	5
4	F, 86	23.0	CI(07/15/23), Paroxysmal AT, HTN	Intact	1	1	1	2	N/A	5
5	M, 73	18.9	S-ICH(05/06/23), HTN, Both knee OA	Delayed	3	1	1	1	N/A	5
6	M, 77	20.8	CI(06/16/23), HTN, DM	Delayed	1	1	8	5	N/A	4
7	M, 59	17.9	SAH(02/06/23), TB (NTM)	Delayed	1	6	5	8	N/A	3
8	M, 85	21.3	CI(06/21/23), A-Fib	Delayed	2	2	2	5	N/A	5
9	M, 59	21.8	S-IVH(05/29/23), TIA, HTN	Delayed	1	8	N/A	N/A	N/A	2
10	M, 67	20.7	CI(09/09/22), Aspiration Pneumonia,	Delayed	3	2	6	6	5	2
11	F, 70	15.6	CI(, Hemiplegia, DM, Alzheimer' s	Delayed	1	1	5	5	N/A	4
12	M, 78	23.0	CI(06/18/23), Agina stent, HTN	Delayed	2	1	2	8	N/A	5
13	M, 59	22.1	S-IVH(05/29/23), TIA, Cataract Rt., HTN	Delayed	1	1	7	N/A	N/A	4
14	F, 77	21.2	S-SAH(02/19/23), S-ICH(02/19/23), DM	Delayed	1	1	2	8	N/A	3
15	F, 70	27.0	CI(08/08/23), SAH, NPH, DM, HT	Delayed	3	2	3	2	1	4
16	F, 90	29.3	CI(07/03/23), Dementia, Capal Tunnel	Delayed	2	N/A	3	N/A	N/A	2
17	M, 74	22.6	CI(08/14/23), BA occlusion	Delayed	7	N/A	N/A	N/A	N/A	1
18	M, 71	24.1	CI(07/10/23), Lt. hemiplegia	Delayed	3	3	3	5	3	3
Mildly Symptomatic Patients										
19	F, 50	27.0	S-SAH(05/04/23), Aneurysm, HTN	Intact	2	1	1	2	1	5
20	F, 81	23.5	CI(05/03/23), HTN, MDD	Delayed	1	1	1	2	2	5
21	F, 77	22.2	CI(06/05/23), DM, dyslipidemia	Intact	1	1	1	1	1	5
22	F, 80	26.3	S-ICH(05/29/23), SAH, HTN	Intact	1	1	2	2	1	6
23	F, 71	27.9	CI, Paroxysmal AFib	Intact	1	1	1	1	1	5
24	M, 63	17.0	S-SAH(03/14/23), S-ICH, IVH, VP Shunt	Delayed	1	1	1	1	1	4
25	M, 56	18.4	CI(05/08/23), Rt. hemianopsia	Intact	1	1	1	1	1	7
26	M, 74	22.2	CI(06/08/23), DM, HTN	Delayed	1	1	2	2	1	5
27	F, 72	25.3	S-ICH(07/16/23), NHL, HTN	Intact	1	1	1	1	1	6
28	M, 69	22.5	S-IVH(07/17/23), HTN	Intact	1	1	1	1	1	5
29	M, 64	24.5	CI(08/08/23), PACI MCA, Rt. ICA	Intact	1	1	1	1	1	7
30	F, 78	25.8	CI(08/14/23), HTN, Both TKR	Intact	1	1	1	1	1	6

The ASHA NOMS (American Speech-Language-Hearing Association National Outcome Measurement System) swallowing levels scale is a metric composed of stages 1 to 7, used to assess patients' swallowing ability quantitatively. Level 1 indicates the most difficult state of swallowing, where nothing can be safely consumed orally, while Level 7 represents an optimal state where swallowing occurs most smoothly. The abbreviation for each disease is CI: Cerebral Infarction; CKD: Chronic kidney disease; HTN: Hypertension; DM: Diabetes mellitus; OA: Osteoarthritis; SAH: Subarachnoid hemorrhage; TB: Tuberculosis; NTM: Nontuberculous mycobacteria; TIA: transient ischemic attack; VP: ventriculoperitoneal; BA: Basilar artery; NPH: Normal pressure hydrocephalus; NHL: Non-Hodgkin lymphoma; PACI: Partial anterior circulation infarct; MCA: middle cerebral artery; ICA: internal carotid artery; TKR: Total knee replacement; S-ICH: symptomatic intracerebral hemorrhage; S-IVH: Spontaneous Intraventricular Hemorrhage.

Table S3. Machine learning layer information for swallowing status classification. Detailed information about the layers of the proposed network for swallowing status classification.

Layer	Output	# filter	Kernel size	Option
Input	500×3			
Conv1D-1_1	500×80	80	5	Padding = Same<br>Activation = PReLU
Conv1D-1_2	500×16	16	4	Padding = Same<br>Activation = PReLU
BatchNorm	500×16			
MaxPool1D-1	125×16		4	
Conv1D-2_1	125×48	48	3	Padding = Same<br>Activation = elu
Conv1D-2_2	125×16	16	4	Padding = Same<br>Activation = PReLU
BatchNorm	125×16			
MaxPool1D-2	31×16		4	
Conv1D-3_1	31×112	112	3	Padding = Same<br>Activation = PReLU
Conv1D-3_2	31×16	16	4	Padding = Same<br>Activation = PReLU
BatchNorm	31×16			
MaxPool1D-3	7×16		4	Stride: 3×1;<br>Mode = Same
Bi-LSTM	7×20	10		Return_sequences = True<br>Activation = tanh
Dense-1	140			Activation = PReLU
Dense-2	512			Activation = PReLU
Dropout	256			$\rho = 0.20$
Dense-3	256			Activation = PReLU
Flatten_final	7			Activation = softmax
Output	7			

Table S4. Hyperparameter tuning information for swallowing status classification. This table details the hyperparameter tuning conducted using the RandomSearch algorithm from Keras Tuner, aimed at optimizing the validation accuracy of our CNN-LSTM model. The tuning employed a uniform distribution to sample hyperparameters across defined search spaces, which included a range of filters, kernel sizes, unit numbers, and dropout rates. A total of 50 different hyperparameter combinations were evaluated, with each combination undergoing two trials to ensure the reliability of the results. The table lists the optimal values identified for each hyperparameter.

Layer	Type	Search Space	Tuned Value
Conv1D-1_1	Filter	32 to 128 (step of 16)	80
Conv1D-1_1	Kernel	{3,5}	5
Conv1D-2_1	Filter	32 to 128 (step of 16)	48
Conv1D-2_1	Kernel	{3,5}	3
Conv1D-3_1	Filter	32 to 128 (step of 16)	112
Conv1D-3_1	Kernel	{3,5}	3
Dense-1	Unit	32 to 512 (step of 32)	140
Dense-2	Unit	32 to 512 (step of 32)	512
Dropout	Unit	0.0 to 0.5 (step of 0.05)	0.2
Dense-3	Unit	32 to 512 (step of 32)	256
Learning rate	Value	{0.01, 0.001, 0.0001}	0.0001
